# Draft genome of *Sagittula* sp. SSi028 isolated from the brown alga *Sargassum ilicifolium* in Singapore

**DOI:** 10.1128/mra.00269-25

**Published:** 2025-05-28

**Authors:** Sujatha Srinivas, Jessica A. Taylor, Su Xuan Gan, Prasha Maithani, Joao P.A. Pereyra, Rebecca J. Case

**Affiliations:** 1 Singapore Center for Environmental Life Sciences Engineering (SCELSE), Nanyang Technological University, Nanyang, Singapore; 2 School of Biological Sciences (SBS), Nanyang Technological University, Nanyang, Singapore; Montana State University, Bozeman, Montana, USA

**Keywords:** brown alga, *Sargassum*, *Sagittula*, roseobacters, quorum sensing, DMSP, lignin, Singapore

## Abstract

*Sagittula* sp. SSi028 was isolated from the globally distributed brown alga *Sargassum ilicifolium*, which is the most abundant seaweed in Singapore. Its genome contains genes for two dimethylsulfoniopropionate assimilation pathways, degradation of lignin-related aromatic compounds, biosynthesis of multiple vitamin Bs, and *luxI-luxR* homologs associated with quorum sensing.

## ANNOUNCEMENT

The genus *Sagittula* belongs to the abundant, ubiquitous, and metabolically versatile marine family, *Roseobacteraceae* (1, 2). Roseobacters are often associated with eukaryotic hosts, particularly dimethylsulfoniopropionate (DMSP)-producing algae (3, 4). Most roseobacters have quorum sensing (QS) systems significant in host interactions (5, 6). *Sagittula* spp. have algicidal activity and are effective in lipid recovery (7) and controlling harmful algal blooms (8). They are implicated in carbon cycling (9) through their lignolytic capabilities (1).

Thalli (5–6 cm long × 2.5–3 cm wide) from three submerged *Sargassum ilicifolium* individuals were collected from St. John’s Island, Singapore (1.222353 N 103.848702 E) during low tide in August 2022 and transported to the laboratory in sterile seawater. Thallus was heat treated at 55°C for 6 min before stamping (10) onto marine broth agar plates (37.4 g/L Difco 2216, 15 g/L agar). Plates were incubated for 24 h at 30°C, and individual colonies were subcultured to ensure purity. For DNA extraction, bacterial biomass from plates and liquid cultures (160 rpm) was combined and washed with 1× PBS.

DNA was extracted from 35 mg (± 10 mg) cell pellets with DNeasy Blood and Tissue Kit (Qiagen, Hilden, Germany), with the addition of RNase A, and purified with Zymo genomic DNA Clean and Concentrator-10 kit (Zymo Research, Irvine, USA). Library preparation used the TruSeq Nano DNA kit (Illumina, San Diego, USA), and 150 bp paired-end sequence libraries were generated using the Illumina HiSeq XTM platform (Illumina, USA). DNA library preparation and sequencing were performed by the SCELSE Sequencing Facility, NTU.

Default parameters were used for all software unless specified. Paired-end reads were trimmed using Trimmomatic v0.39 (11). The genome was assembled using the Shovill pipeline v1.1.0 (https://github.com/tseemann/shovill) and St. Petersburg genome assembler (SPAdes) v3.15.5 (12). Genome annotation was performed using PGAP v6.7 (13). The assembly was checked for plasmids with PlasmidFinder v2.0.1 (14), but no plasmids were found.

Isolate was classified using Genome Taxonomy Database Toolkit v1.7.0 based on phylogenomic analysis and average nucleotide identity (ANI) comparisons (15), with the closest taxonomic placement to *Sagittula marina* (GCF_014196795.1). Phylogenetic relatedness between the isolate and all publicly available *Sagittula* reference genomes was determined using Orthologous Average Nucleotide Identity Tool v0.93.1 (16) and Genome-To-Genome Distance Calculator v3.0 (17) to calculate ANI and DNA-DNA hybridization (DDH) scores, respectively. Results showed <95% ANI and <70% DDH with all reference genomes, which is below the species threshold (18) (76.95% ANI and 20.4% DDH to *Sagittula marina*).

The genome encodes genes for DMSP utilization via cleavage (*dddW*) (19) and demethylation (*dmdB* and *dmdC*) (20) pathways and gene homologs (*pcaB*, *pcaD*, and *pcaF*) for the degradation of aromatic compounds via β-ketoadipate pathway (21), supporting the potential lignolytic capability of *Sagittula* sp. SSi028. Like other algal symbionts, this isolate genome has homologs for vitamins B_1_, B_2_, B_5_, B_6_, and B_12_ biosynthesis genes (22). The presence of one *luxI* homolog, three *luxR* homologs, and an acyl-homoserine lactone acylase and acyl-homoserine lactonase indicates an acyl-homoserine lactone-based QS and quenching system. These features suggest a symbiotic interaction with its host, through the production and utilization of metabolites and signals.

**TABLE 1:** Genome features and GenBank accession number for *Sagittula* sp. SSi028

Isolate	Genome size (bp)	No. of CDS^a^	No. of rRNAs	No. of tRNAs	G + C content (%)	No. of contigs	*N*_50_ (bp)	*L*_50_	Assembly accession no.	SRA accession no.	No. of reads	Average read length (bp)
SSi028	3,913,045	3,649	8	50	61.5	80	436,642	4	JBLZNC000000000	SRR32627963	9,682,878	151

^a^ CDS, coding DNA sequences.

## Data Availability

The SRA data were deposited to NCBI, and the complete genome sequence was deposited to GenBank under the accession numbers listed in Table 1.
